# Neurologic Sequela of COVID-19: Guillain-Barré Syndrome Following Johnson & Johnson COVID-19 Vaccination

**DOI:** 10.7759/cureus.24252

**Published:** 2022-04-18

**Authors:** Octavio Carranza, Denis Babici, Sadia Waheed, Fawad Yousuf

**Affiliations:** 1 Neurology, Florida Atlantic University Charles E. Schmidt College of Medicine, Boca Raton, USA; 2 Neurology, Marcus Neuroscience Institute, Boca Raton Regional Hospital, Boca Raton, USA

**Keywords:** janssen covid-19 vaccine, covid-19 vaccine related adverse events, johnson and johnson vaccine, guillain- barré syndrome, covid-19 vaccine

## Abstract

Guillain-Barré syndrome after Pfizer and Astra-Zeneca vaccinations against COVID-19 has been previously reported in the literature. The objective of this study was to report the first case of Guillain-Barré following the COVID-19 vaccination with the Johnson & Johnson COVID-19 vaccine. We report the case of a 53-year-old female who presented to the Emergency Department complaining of bilateral lower extremity weakness, paresthesias, and gait difficulties 14 days after having received the Johnson & Johnson COVID-19 vaccination. MRI of the lumbar spine with and without contrast revealed enhancement of the cauda equina nerve roots suggestive of acute inflammatory demyelinating polyradiculoneuropathy. Cerebrospinal fluid (CSF) analysis reported mildly elevated protein low white blood cells (WBCs). Ganglioside (GM1 and GQ1b) antibodies were reported as negative. After intravenous immunoglobulin (IVIg), the patient had significant improvement in her weakness and paresthesia and was discharged home. The case was reported to the Centers for Disease Control and Prevention (CDC) Vaccine Adverse Event Reporting System. Guillain-Barré syndrome after COVID-19 immunization remains a rare complication. A clear mechanism of disease has not been clarified; however, it is believed that there could be some type of molecular mimicry between the spike glycoprotein produced with the help of the vaccines and proteins in the myelin sheath.

## Introduction

The first case of novel severe acute respiratory syndrome coronavirus 2 (SARS-CoV-2) was reported in Wuhan, China, in December 2019 [1], and in a few months became a global pandemic. Patients with SARS CoV-2 infection can present with milder symptoms, including sore throat, headache, dry cough, fever, fatigue, mild shortness of breath, and diarrhea. However, more severe diseases can present with acute respiratory distress syndrome, coronavirus 19 (COVID-19) pneumonia, and later coagulopathy [2]. COVID-19 has been reported to manifest with neurologic complications, including post-infectious Guillain-Barré syndrome (GBS) [3], encephalopathy [4], and seizures [5].

The first correlation between vaccination and GBS was made in the year 1976-1977 after receiving influenza vaccination [6]. Up to this day, this correlation remains controversial. COVID-19 continues to be a major pandemic concern around the world to date. Multiple vaccination strategies have been implied to control the pandemic and to limit the spread of the infection. To this date, there are case reports that associate the Pfizer COVID-19 vaccine with Guillain-Barré syndrome. However, to the best of our knowledge, GBS has not been reported after receiving Johnson & Johnson (J&J) vaccine. Here we report the first case of GBS following the J&J vaccine.

## Case presentation

**Figure 1:**
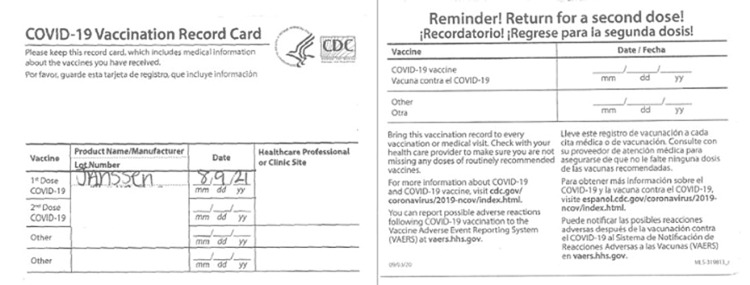
COVID-19 vaccination card

A 53-year-old female with a past medical history of hypothyroidism and Bell's palsy presented to the Emergency Department with complaints of a two-week history of progressive lower extremity weakness, paresthesias, and difficulty walking that started after receiving the COVID-19 J&J vaccination 14 days before her presentation (Figure 1).

The patient reported that minutes after receiving the vaccine, she started feeling the development of a rash in her palms, followed by the appearance of numbness and tingling in the distal left upper extremity. Hours after, the numbness progressed slowly in an ascending fashion through the left lower extremity, and overnight it extended to the contralateral lower extremity and left side of the face. The numbness was later accompanied by lower thoracic and lumbar pain.

Because of her symptomatology, she went to see her primary care provider, who prescribed her a short course of steroids and valganciclovir after suspecting a new episode of peripheral facial palsy. Due to the lack of improvement in her symptoms and a near syncopal episode at her home, she decided to come to our Emergency Department. At the time, the neurological examination revealed mild left-sided weakness in the left side of her body when compared to the contralateral side and intact reflexes throughout. MRI of the brain without contrast done during this first encounter was negative for any acute intracranial pathology, and she was advised to continue to follow up with neurology as an outpatient and to consider electromyography and nerve conduction studies.

Five days later, the patient presented again to our institution due to progressive proximal weakness, paresthesias, and difficulty standing up from a chair and walking. At the time, she denied any dyspnea, dysphagia, or diurnal variation in the weakness. On neurological exam, her mental status was alert, attentive, and oriented to person, place, and time. The speech was clear and fluent, with a good repetition, comprehension, and naming. She recalled 3/3 objects after five minutes. Cranial Nerves were intact, with full visual fields for confrontation. Pupils were round and reactive to light and accommodation. Extraocular movements were normal, and no nystagmus or diplopia was apparent. Her facial sensation was intact in all three branches of the trigeminal nerve bilaterally; the face was symmetric with normal eye closure and smile, the palate elevated symmetrically, phonation was normal, head-turning and shoulder shrug were intact, tongue protruded towards the midline, with normal movements and no atrophy. 

**Figure 2:**
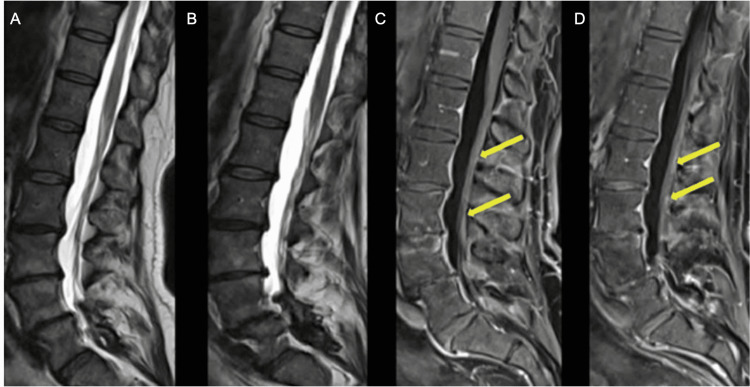
MRI of the lumbar spine with and without contrast A and B: Sagittal T2 sequence demonstrating T2 hyperintensities in cauda equina roots. C and D: Sagittal T1 post-contrast sequence demonstrating Gadolinium enhancement of the cauda equina roots.

On motor exam, she was noted to have proximal weakness 4/5 in hip flexors and hip extensors bilaterally, 4/5 knee extensors and flexors, 4/5 plantar flexion, 4/5 dorsiflexion bilaterally, 4/5 shoulder abductors and adduction, 4/5 elbow extensor and flexors. Reflex examination was pertinent for 2+ reflexes in the biceps and triceps 2+, knees, and 1+ Achilles’ tendon reflex bilaterally. Sensory examination revealed normal sensation to light touch, pinprick, position sense, and vibration in fingers and toes. Coordination was normal, with rapid alternating movements intact. Gait was steady with normal steps, base arm swing, and turning. Heel and toe walking and tandem gait was normal. An MRI of the lumbar spine with and without contrast revealed subtle enhancement of the cauda equina nerve roots without overt thickening, suggestive of acute inflammatory demyelinating polyradiculoneuropathy (AIDP) (Figure 2). 

She was admitted to the hospital out of suspicion of probable Guillain-Barré Syndrome. Due to this concern, the patient was started with intravenous immunoglobulin. Subsequent Lumbar puncture and CSF analysis reported a clear appearance, a very mild elevated protein with a slightly elevated mononuclear pleocytosis, and normal glucose levels (Protein 47 mg/dL, Albumin 25 mg/dL, Glucose 63 mg/dL, red blood cells [RBCs] 0/mcL, WBC 1/mcL 100% mononuclear, venereal disease research laboratory test [VDRL] non-reactive, lactate dehydrogenase [LDH] 23 IU/L). GM1 and GQ1b antibodies were reported as negative. (Table 1)

**Table 1:** Cerebrospinal fluid analysis

Laboratory test in CSF	Patient results	Reference Range
Protein	47 mg/dL	15-45 mg/dL
Albumin	25 mg/dL	0-35 mg/dL
Glucose	63 mg/dL	40-75 mg/dL (>60% Serum Glucose)
RBCs	0/mcL	0 cells/microLiter
WBCs	1/mcL	0-4 cell/microLiter
LDH	23 UI/L	<40 UI/L

The patient started to feel improvement in her weakness from day two of IVIg and continued to receive this treatment for a total of five days without any complications or adverse effects. Her physical exam on the day of discharge showed 5/5 proximal and distal strength in all her extremities and normal deep tendon reflexes. She was discharged home with home health care, with outpatient physical therapy, and was scheduled for follow-up as an outpatient in our neurology clinic. The Centers for Disease Control Vaccine Adverse Event Reporting System (VAERS) was notified about this abnormal reaction.

## Discussion

The first report of Guillain-Barré syndrome following vaccination for COVID-19 was reported in February of 2021 after receiving the Pfizer mRNA COVID-19 vaccination [7]. Since then, four additional GBS cases have been reported in the literature after receiving the Pfizer vaccine [8-11]. The AstraZeneca immunization has also been mentioned to have a similar adverse effect in the other four case reports [12-15]. To this date, the Vaccine Adverse Event Reporting System from the CDC has accumulated 575 reports of Guillain-Barré syndrome following COVID-19 immunizations [16]. To our knowledge, this is the first case report in the literature of Guillain-Barré Syndrome following a J&J vaccine.

The United States (US) Food and drug administration (FDA) approved the J&J vaccination in February 2021. A few reported common adverse effects after receiving the J&J vaccine are injection site pain, headache, fatigue, myalgia, nausea, fever, injection site erythema, and injection site swelling. In July, the FDA issued a warning for Guillain-Barré syndrome after 42 days of receiving a J&J vaccination. However, it is important to mention that the period for maximal efficacy after immunization with this vaccine is approximately 14 days which made the association questionable. This vaccine has also been associated with potential hypercoagulability and cerebral sinus thrombosis with thrombocytopenia; however, as expected, these severe adverse reactions appear to present very rarely. 

The J&J vaccine utilizes a modified non-replicating, viral vector to transport COVID-19's spike protein genetic code to the patient´s immune cells, which are in charge of creating antibodies and stimulating memory cells to react against the virus. [17]. This vaccine demonstrated 75% overall efficacy against COVID-19.

One of the hypothesized mechanisms leading to peripheral nervous system involvement and damage after receiving an mRNA vaccine is said to be molecular mimicry between spike glycoprotein synthesized after the mRNA is introduced into the body and the neuronal myelin sheath; however, up to date, the exact mechanism between GBS and COVID-19 vaccination remains controversial [18]. This case report adds to the literature evidence for the possible development of AIDP following immunization with the third type of vaccine available in the USA, the Janssen vaccine.

## Conclusions

To the best of our knowledge, we report the first real-life case of Guillain-Barré syndrome associated with the Johnson & Johnson COVID-19 vaccine. While there is insufficient evidence to establish a temporal correlation, we strongly believe that physicians should remain watchful, have a high degree of suspicion, and be able to recognize neurological complications associated with the COVID-19 vaccination. Early diagnosis and subsequent management can prevent disability. The risk of neurological complications after the COVID-19 vaccination is low, and the benefits of the vaccination outweigh the risks and side effects. 
